# Immunological detection of occult blood in faeces in colorectal cancer.

**DOI:** 10.1038/bjc.1984.268

**Published:** 1984-12

**Authors:** D. J. St John, F. A. Macrae


*Br. J. Cancer* (1984), **50**, 853–854

### Letters to the Editor

### Immunological detection of occult blood in faeces in colorectal cancer

**Sir** – The above paper by Turunen *et al.* (1984) provides interesting data about the relative sensitivities of FECA-EIA, an immunological test for human haemoglobin (Hb), and four guaiac tests for the detection of faecal occult blood. The authors also present data on blood loss in 19 patients with colorectal cancer. The observation that blood loss from cancers in the R. hemicolon is greater than that from cancers in the L. hemicolon and rectum confirms our findings (Macrae & St. John, 1982). However the authors draw the additional conclusion that blood loss is related to tumour stage, incorrectly stating that this is similar to our findings.

The issue about a relationship between blood loss and tumour stage is important as it has implications for the likely situation in early cancers that may be diagnosed in screening programmes.

We have reviewed our data based on 380 twenty-four hour faecal samples obtained from 46 patients with colorectal cancer. For the purposes of comparison, the results have been grouped as in the article by Turunen *et al.* (Table). Separate analyses of variance were performed on log-transformed measurements of mean daily blood loss and mean faecal Hb concentration to assess their relationship with tumour site and stage. Blood loss ($P<0.001$) and Hb concentration ($P<0.01$) varied significantly with site of tumour. Stage had no effect after fitting site. Furthermore, there was no interaction between site and stage. In claiming a correlation between blood loss and tumour stage, it appears that the authors have failed to allow for the effect of site of tumour on the results.

**Table** Relationship between geometric mean levels of blood loss and tumour site and stage in 46 patients with colorectal cancer.

| | *No. of Patients* | *Blood loss ml day$^{-1}$* | *Blood loss mg Hb g$^{-1}$* |
|---|---|---|---|
| R. Hemicolon | | | |
| Dukes A & B | 8 | 12.5 | 19.5 |
| Dukes C & D | 2 | 3.0 | 4.5 |
| L. Hemicolon | | | |
| Dukes A & B | 10 | 1.9 | 3.1 |
| Dukes C & D | 8 | 1.9 | 2.9 |
| Rectum | | | |
| Dukes A & B | 10 | 2.0 | 2.8 |
| Dukes C & D | 8 | 1.5 | 2.3 |

Yours etc.

D. James B. St. John,
F.A. Macrae.
Department of Gastroenterology,
The Royal Melbourne Hospital,
Melbourne 3050,
Australia.

**References**

MACRAE, F.A. & ST. JOHN, D. J. B. (1982). Relationship between patterns of bleeding and Hemoccult sensitivity in patients with colorectal cancers or adenomas. *Gastroenterology* **82,** 891.

TURUNEN, M. J. LIEWENDHAL, K., PARTANEN, P., ADLERCREUTZ, H. (1984). Immunological detection of faecal occult blood in colorectal cancer. *Br. J. Cancer,* **49,** 141.

*Dr Turunen & colleagues reply:*

**Sir** – It is possible that the significant correlation between faecal blood loss and tumour stage in colorectal cancer observed by us might reflect an effect of tumour site on the results, as we have also observed that patients with cancer in the right hemicolon excrete more blood than those with cancer in the left hemicolon and rectum. However, our results from 19 patients, grouped as in the Table presented by St. John and Macrae, do not support such a conclusion. Admittedly, the number of observations per group is small and not suitable for statistical analysis. We therefore welcome the report of St. John and Macrae on 46 patients. Even larger materials will be needed for ultimate conclusions on this controversial and important issue. We apologize to Drs. St. John and Macrae for incorrect citation of their earlier report and for any inconvenience this might have caused them.

**Table** Relationship between geometric mean levels of blood loss and tumour site and stage in 19 patients with colorectal cancer.

| | *No. of Patients* | *Blood loss ml day$^{-1}$* | *Blood loss mg Hb g$^{-1}$* |
|---|---|---|---|
| R. Hemicolon | | | |
| Dukes A & B | 2 | 9.6 | 7.2 |
| Dukes C & D | 4 | 25.8 | 23.6 |
| L. Hemicolon | | | |
| Dukes A & B | 6 | 2.7 | 4.3 |
| Dukes C & D | 2 | 4.7 | 6.9 |
| Rectum | | | |
| Dukes A & B | 3 | 1.7 | 1.6 |
| Dukes C & D | 2 | 7.1 | 4.8 |

Yours etc.

M.J. Turunen, K. Liewendahl, P. Partanen, H. Adlercreutz
Second Dept. of Surgery,
Dept. of Clinical Chemistry,
Helsinki University Central Hospital and
Labsystems Research Lab., Labsystems Corp.,
Helsinki, Finland.


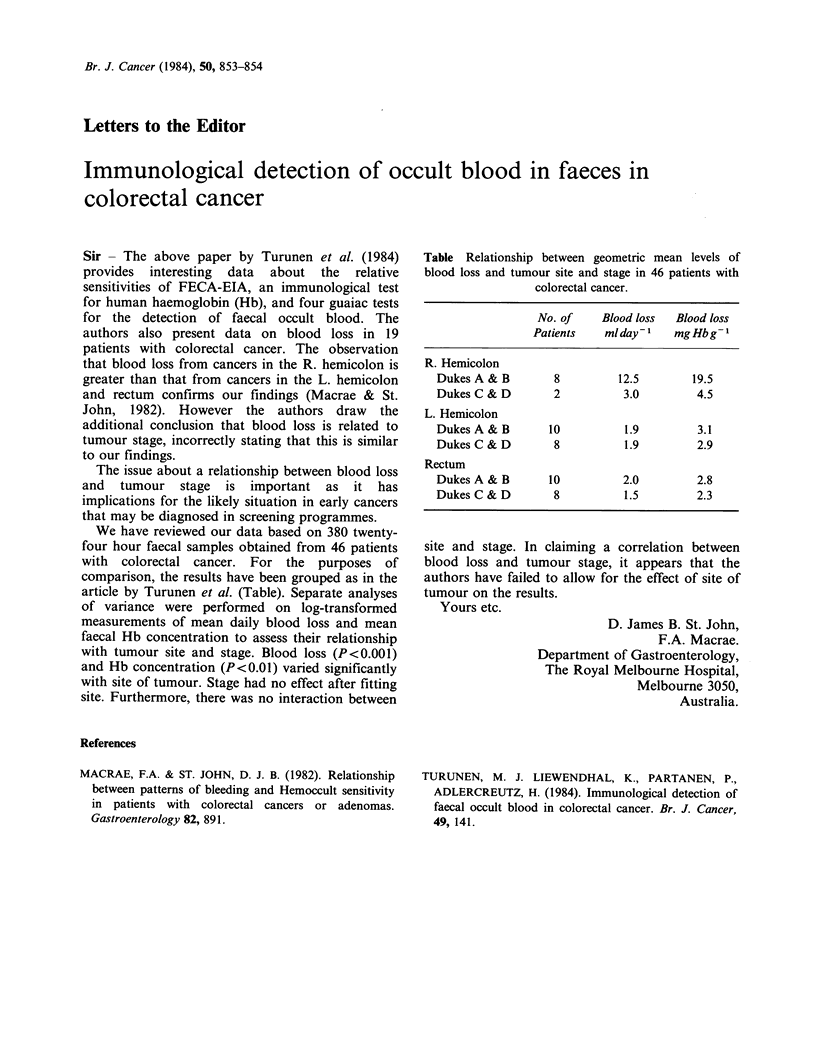

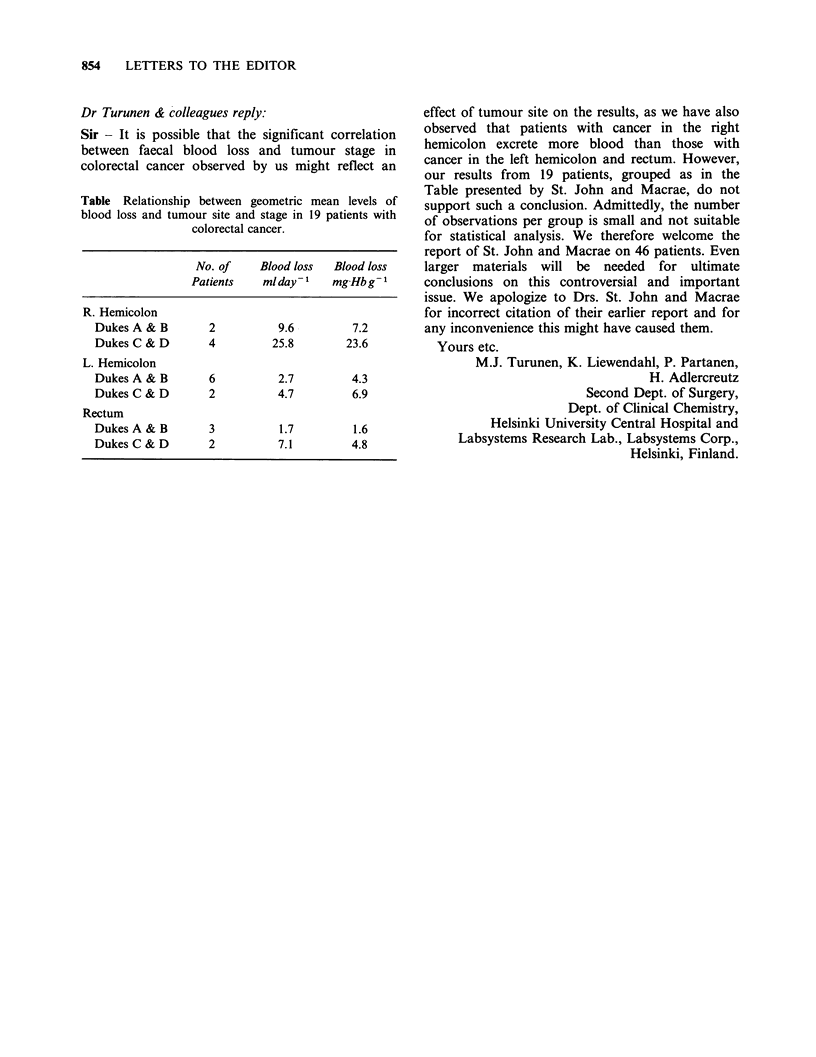


## References

[OCR_00082] Macrae F. A., St John D. J. (1982). Relationship between patterns of bleeding and Hemoccult sensitivity in patients with colorectal cancers or adenomas.. Gastroenterology.

[OCR_00090] Turunen M. J., Liewendahl K., Partanen P., Adlercreutz H. (1984). Immunological detection of faecal occult blood in colorectal cancer.. Br J Cancer.

